# Perioperative Inflammatory Response and Cancer Recurrence in Lung Cancer Surgery: A Narrative Review

**DOI:** 10.3389/fsurg.2022.888630

**Published:** 2022-07-11

**Authors:** Hoon Choi, Wonjung Hwang

**Affiliations:** Department of Anesthesia and Pain Medicine, Seoul St. Mary's Hospital, College of Medicine, The Catholic University of Korea, Seoul, Republic of Korea

**Keywords:** inflammation, lung cancer, neoplasm metastasis, neoplasm recurrence, perioperative management

## Abstract

While surgical resection is the gold standard treatment for solid tumors, cancer recurrence after surgery is common. Immunosurveillance of remnant tumor cells is an important protective mechanism. Therefore, maintenance of anti-tumor cell activity and proper levels of inflammatory mediators is crucial. An increasing body of evidence suggests that surgery itself and perioperative interventions could affect these pathophysiological responses. Various factors, such as the extent of tissue injury, perioperative medications such as anesthetics and analgesics, and perioperative management including transfusions and methods of mechanical ventilation, modulate the inflammatory response in lung cancer surgery. This narrative review summarizes the pathophysiological mechanisms involved in cancer recurrence after surgery and perioperative management related to cancer recurrence after lung cancer surgery.

## Introduction

Lung cancer is the second most common type of cancer in the world and the leading cause of cancer-related deaths (1). Non-small-cell lung cancer (NSCLC) accounts for up to 85% of all lung cancers, and like most other solid cancers, is primarily treated with surgical resection for curative intent. Despite curative resection, 30%–55% of NSCLC patients experience recurrence and metastasis; the median survival time of patients with NSCLC recurrence is only about 21 months (2, 3). Even after complete surgical resection, microscopic tumor cells may remain, which increases the risk for local recurrence and distant metastasis through circulating tumor cells (CTCs) that disseminate in the bloodstream or the lymphatic system (4).

Surgery elicits a stress response that results in a catecholamine surge and activation of physiologic responses that promote wound healing and organ recovery. However, these physiologic responses, including the inflammatory response and angiogenesis, may contribute to cancer recurrence and metastasis (5). For example, perioperative inflammation and subsequent immunosuppression inhibit natural killer (NK) cell and T lymphocyte activity, which is critical for CTC detection and clearance (6). In this regard, evidence is accumulating that operative and anesthetic techniques may influence the effect of surgery on inflammatory response and cancer recurrence. This review summarizes the pathophysiological mechanisms involved in cancer recurrence after surgery and perioperative management related to cancer recurrence after lung cancer surgery.

## Material and Methods

We extracted the most recent evidence from various databases, including PubMed, EMBASE, Web of Science, Google Scholar, and Cochrane Library databases. A literature search was performed using the following keywords: anesthesia, anesthetics, lung cancer, cancer recurrence, metastasis, and inflammation. All retrieved articles and relevant reviews were manually searched to find other potentially eligible studies. There was no restriction for the article type. Appropriateness for inclusion was determined by the authors to include a wide and unbiased range of relevant and recent studies.

## Links Between Inflammatory Response and Cancer Progression

### Inflammation and Cancer Recurrence

After surgical resection, tumor cells can disseminate in the peripheral blood as CTCs or propagate to the bone marrow or lymph nodes as disseminated tumor cells (DTCs) (5–7). The presence of CTCs does not entirely represent metastasis or recurrence. The host immune system generally detects and eliminates them. However, surgical stress can induce remnant cancer cells with complex involvements of sympathetic, inflammatory, and immune systems. In lung cancer surgery, CTC numbers are increased following surgery (8, 9), and are associated with cancer recurrence (10).

Cancer cells, inflammatory cells, immune cells, and stromal elements within the tissue interact with each other in a complex and dynamic way (5–7). This is called a “tumor microenviroment (TME)” that determines the potential for tumor metastasis. In normal conditions, the lack of extracellular matrix support, damage by shear stress, and immune surveillance hampers CTC survival and colonization. However, surgical intervention and tissue trauma easily disrupt TME and promote spread of residual cancer cells. Cancer recurrence occurs from remnant tumor cells at surgical sites in four stages (5, 6). First, remnant tumor cells acquire fibroblast-like properties such as motility, invasiveness, and exudation. This is called the “epithelial-mesenchymal transition”. Second, tumor cells invade the basement membrane, lymphatics, and blood vessels. Third, CTCs mitigate or survive in the circulatory system and metastasize to distant sites. Finally, single progenitor cells interact with stromal and inflammatory cells to proliferate. The inflammatory microenvironment plays a crucial role in the modulation of these steps. For example, NK and cytotoxic T (Tc) cell activity decrease after surgery, resulting in remnant tumor cells. Excessive secretion of growth factor and enzymes is harmful because they increase invasiveness and permeability of the tumor cells.

**Figure 1:**
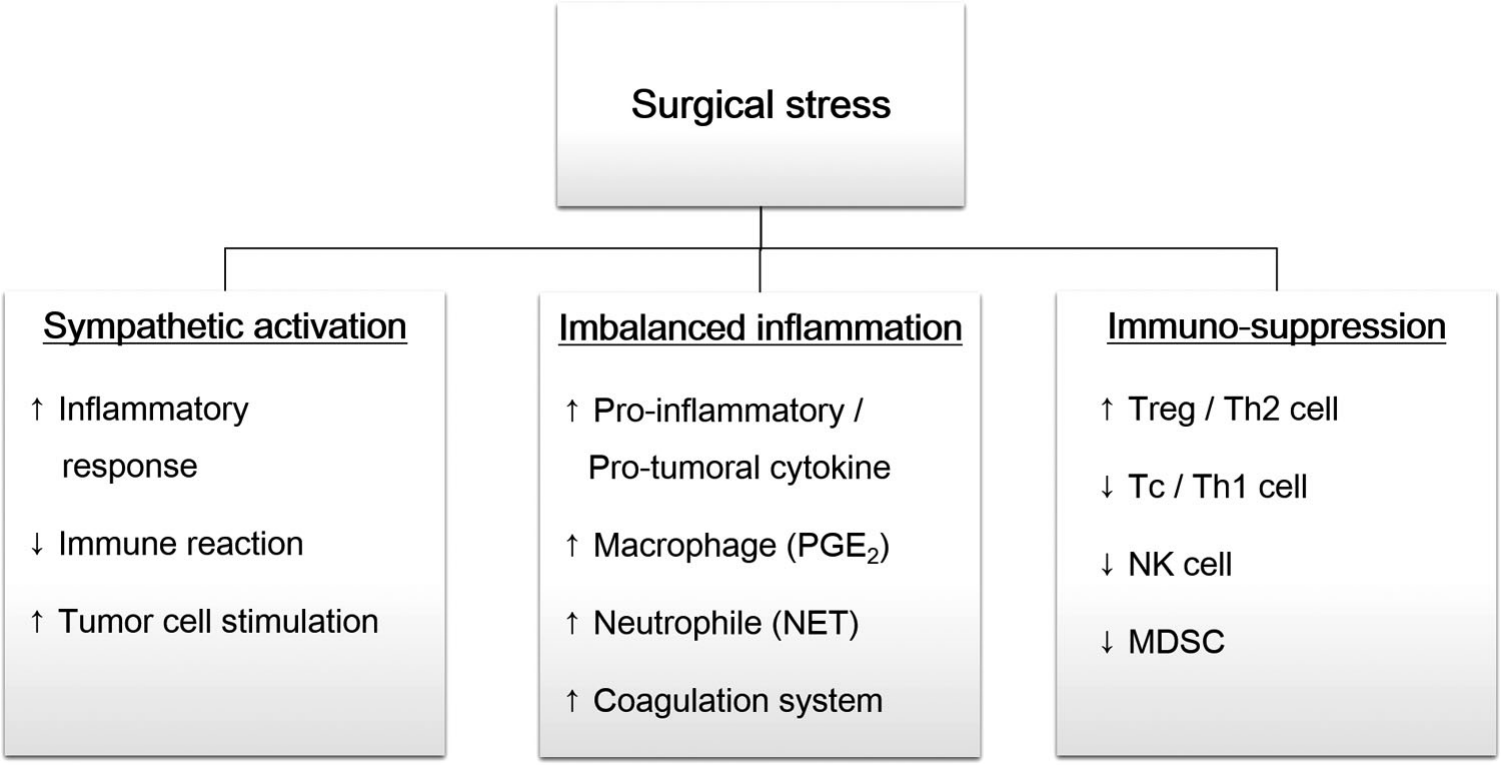
Overview of surgical stress response. Surgical trauma induce sympathetic activation, followed by imbalanced inflammation and immunosuppression. These changes promote remnant tumor cells survival and distant metastasis. PGE2, prostaglandin E2; NET, neutrophil extracellular traps; Treg, regulatory T cell, Th, helper T cell; Tc, cytotoxic T cell; NK cell, natural killer cell; MDSC, myeloid-derived suppressor cell.

Surgery-induced stress response can promote tumor cell shedding by sympathetic activation, inflammatory imbalance, and immunosuppression (Figure 1).

#### Sympathetic Activation

Sympathetic activation triggers inflammatory response and promotes tumor cell growth. Tumor cell excision stimulates the hypothalamic-pituitary-adrenal axis and the sympathetic nervous system to release cortisol and catecholamines (11, 12). These factors increase pro-inflammatory cytokines (e.g., interleukin [IL]-6 and IL-8) and immunosuppressive cytokines (e.g., IL-4, IL-10, TGF- β, VEGF). Activated sympathetic system suppress NK and Tc cell activity, both of which are the main cells of immunosurveillance. They also stimulate helper T (Th)2 and regulatory T (Treg) cell proliferation. In addition, catecholamines directly act on tumor cells *via* the *β*-receptors. Activated *β*-receptors increase pro-inflammatory cytokines, vascular endothelial growth factor (VEGF), and matrix metalloprotease (MMP), which promotes tumor cell mobility and invasion (13).

#### Imbalanced Inflammatory Response

Initial tissue injury involves the release of humoral factors, resulting in the recruitment and activation neutrophils, macrophages, monocytes, and fibroblasts (5, 6, 14). Recruited inflammatory cells release more pro-inflammatory cytokines, including IL-1, IL-6, tumor necrosis factor (TNF)-α, and neutrophil extracellular traps (NET). These mediators increase the Th1/Th2 ratio and the secretion of interferon (IFN)-γ and IL-2 with anti-inflammatory and anti-tumoral effects.

Unfortunately, an imbalance between the pro- and anti- inflammatory responses can lead to the dysregulation of cellular immunity and subsequent immunosuppression. Macrophages and neutrophils, the main cells of the inflammatory response, continuously secrete IL-1, TNF-α, VEGF, and MMP, which contribute to tumor progression (15). Neutrophils can form a CTC-white blood cell cluster to expand the metastatic potential of CTCs, which promotes cell cycle progression (16). Extravasated neutrophil in the tissues after surgery has been shown to promote tumor capture and growth (17). Circulating neutrophile increase CTCs adhesion to the microvascular endothelium to induce epithelial-mesenchymal transition (18).

Excessive IL-6 stimulates macrophages to release prostaglandin E_2_ (PGE_2_). It increases the growth and motility of cancer cells and can induce angiogenic conversion in various cancers (19). In cases of lung cancer, PGE_2_ levels increase after surgery, which promotes metastasis by upregulation of MMP9 and downregulation of E-cadherin (20). PGE_2_ triggers an immunosuppressive state by increasing cancer-promoting Treg cells, decreasing activated Tc cells, and decreasing the Th1/Th2 ratio toward pro-inflammatory and pro-tumoral effects (21).

NETs released during the inflammatory response also promote cancer recurrence. NETs sequester CTCs and promote metastasis (22). Moreover, sequestered CTCs can trigger NET generation, resulting in tumor-host interaction (23). After surgical stress, NETs promote tumor cell proliferation, adhesion, migration, and invasion by inducing high-mobility group box (HMGB)-1 release, which interacts with platelets through toll-like receptor (TLR)-4 and activates TLR9-dependent pathways (24). In an animal study, NETs have a potential to activate dormant cancer cells following inflammation (25).

Recent trials have demonstrated that the complement system and fibrinogen also play a role in cancer recurrence. The complement proteins C7 and CFH are required for the maintenance of stemness in cancer cells (26). C5a anaphylatoxin promote angiogenesis in the tumor microenvironment (27), while complement system activation inhibits T-cell mediated anti-tumor immunity in lung cancer (28). Activated platelets and fibrin coat the CTCs and protect them from detection and removal by NK cells (29). In addition, platelet-fibrin complex mediate tumor cell adherence to endothelial cells and enhance vascular permeability, releasing mitogenentic and proangiogenetic factor.

#### Immunosuppression

The inflammatory response after surgery may promote systemic immunosuppression. Immunosuppressive microenvironments promote tumor progression and metastasis (30). The postoperative immunosuppression may last for about 2 weeks (31), and peaks at 3 days after surgery (32).

Each T cell population contributes differently to tumor cell survival (33, 34). Tc cells are involved in killing the tumor cells, and Th1 cells regulate the cytotoxic immunity to inhibit tumor progression. Meanwhile, Treg cells inhibit anti-tumor immune responses to create a pro-tumorigenic environment, and Th2 cells stimulate MMP expression, invasive potential, and metastasis. After surgery, Treg and Th2 cells are markedly increased, while Tc and Th1 cells are decreased, which contributes to tumor cell survival to varying degrees (31, 35, 36). In lung cancer surgery, an increase in Treg cells is observed in partial tumor resections, preventing the recruitment of Tc cells to the tumors and promoting recurrence rate (30).

NK cells, a type of cytotoxic lymphocyte, play an important role in tumor immunosurveillance as a major source of IFN-γ (37). Both NK cell toxicity and the secretion of IFN-γ by NK cells are profoundly suppressed perioperatively (38). A significant decline in IFN-γ has been observed after partial lung tumor resections (30). Intra-tumoral NK cell density is related to non-small cell lung cancer prognosis and recurrence (39).

Another mechanism leading to immunosuppression after surgery is the downregulation of chemokine (C-X-C motif) ligand 4 (CXCL4) and the recruitment of myeloid-derived suppressor cells (MDSC) (40). MDSCs trigger tumor progression by modulating the formation of premetastatic niches, and by inducing angiogenesis and tumor cell invasion (41). The number of MDSCs after surgery is related to cancer recurrence and prognosis (42). In lung cancer surgery, human MDSCs expressing CD11b(+), CD33(+), and HLA-DR(−) significantly increase after thoracotomy, and promote angiogenesis and tumor growth (43).

### Postoperative Inflammatory Biomarkers as Prognostic Parameters

Objective evaluation of the inflammatory state after surgery may be useful for early detection of patients with a systemic inflammatory response (44). In addition, considering the association between inflammation and cancer recurrence, an evaluation of the inflammatory state after surgery may identify patients at risk for recurrence (45, 46). Predictive biomarkers commonly used to evaluate the inflammatory state after lung cancer surgery, including acute-phase proteins, complete blood count (CBC)-derived values and cytokines, are summarized in Table 1.

**Table 1:** Common inflammatory biomarkers as prognostic parameters after lung cancer surgery.

Biomarker	Change in response to inflammation	Clinical outcomes (references)
Acute-phase proteins		
C-reactive protein	Increase	Lower OS and DFS (47–50) Higher OS and DFS (51)
Fibrinogen	Increase	Lower OS and DFS (52)
Albumin	Decrease	Lower OS and DFS (53)
Prognostic nutrional index	Decrease	Lower OS and DFS (54)
CBC-derived values		
NLR	Increase	Lower OS and DFS (55)
PLR	Increase	Lower OS and DFS (56)
Cytokines		
Interleukin-6	Increase	Lower DFS (57)
Interleukin-4	Decrease	Lower OS and DFS (58)
MIG	Increase	Lower DFS (59)

*OS, overall survival; DFS, disease-free survival; CBC, complete blood count; NLR, neutrophil-to-lymphocyte ratio; PLR, platelet-to-lymphocyte ratio; MIG, monokine-induced by gamma-interferon.*

In response to inflammatory cytokines produced by local inflammatory cells, the liver produces acute-phase reactants, while the production of other proteins decreases (60). The most commonly used acute-phase proteins are C-reactive protein (CRP), fibrinogen, and albumin (Table 1). Postoperative serum CRP (47–51), fibrinogen (52), and albumin (53, 54) levels are associated with overall survival (OS) and disease-free survival (DFS). In a retrospective study of patients who underwent complete resections of pathological stage I and II NSCLCs, the Cox proportional hazard model revealed perioperative CRP grade as an independent poor prognostic factor for OS (grade 3 vs. grade 0 hazard ratio; HR: 5.05, 95% confidence interval; CI, 1.59, 19.60; *p* = 0.005), and DFS (HR: 3.62, 95% CI, 1.50, 9.33; *p* = 0.004) (47). On the other hand, a retrospective study of patients with resected NSCLC demonstrated that high serum CRP levels, measured on postoperative days 3, were associated with a favorable prognosis (HR: 0.36, 95%: CI, 0.20, 0.65; *p* < 0.001) (51). In a prospective study of patients with stage I–IIIA NSCLCs, serum plasma fibrinogen after surgery was an independent predictor for unfavorable DFS (HR: 3.77, 95% CI, 1.24, 9.87; *p* = 0.009) (52). A retrospective study of stage I NSCLC patients identified postoperative hypoalbuminemia as an independent negative prognostic factor for recurrence (HR: 0.221, 95% CI, 0.077, 0.634; *p* = 0.005) (53).

CBC analysis provides the crude numbers of neutrophils, granulocytes, lymphocytes, and platelets (45, 46). CBC-derived values, such as neutrophil-to-lymphocyte ratio (NLR) and platelet-to- lymphocyte ratio (PLR), have been widely studied (Table 1). A retrospective study of stage I NSCLC patients demonstrated that postoperative NLR was an independent predictor of DFS (HR: 2.435, 95% CI, 1.526, 4.322; *p* = 0.001) and OS (HR: 2.747, 95% CI, 1.668, 4.408; *p* = 0.001) (55). A retrospective study of patients with resectable lung cancer demonstrated that increased postoperative/preoperative PLR was independently associated with poor survival (HR: 1.890, 95% CI, 1.238, 2.887; *p* = 0.003) (56).

Cytokines are a broad category of cell signaling proteins that act as immunomodulators (61). IL-1, IL-8, IL-12, IL-18, TNF-α, IFN-γ, and granulocyte-macrophage colony-stimulating factors (GM-CSFs) are recognized as pro-inflammatory cytokines, whereas IL-4, IL-10, IL-13, IFN-α, and transforming growth factor (TGF)-β are anti-inflammatory cytokines. Interestingly, IL-6 exhibits both pro- and anti-inflammatory properties. Inflammatory cytokines related to lung cancer include IL-1β, IL-4, IL-6, IL-11, IL-12, TNF-α, monocyte chemotactic protein (MCP)-1, and TGF-β (62). Studies that have investigated cytokines as prognostic markers for OS and DFS after cancer surgery are scarce because measuring cytokines requires a complex procedure such as flow cytometry, which is expensive and not routinely performed (46). Postoperative IL-6, IL-4, and IFN-γ changes have been suggested as prognostic markers in lung cancer surgery (Table 1). In a prospective study of patients who underwent curative pulmonary resections for NSCLCs, serum IL-6 levels on postoperative day 1 were significant independent predictors of early postoperative recurrences (Odds ratio; OR: 1.008, 95% CI, 1.003, 1.013; *p* = 0.003) (57). A prospective study of patients who underwent radical surgery for NSCLC demonstrated that patients with postoperative IL-4 abnormalities had significantly greater one- and three-year cumulative relapse frequencies compared to patients with normal IL-4 levels (1-year: 40.00% vs. 15.15%; 3-year: 72.00% vs. 33.33%; *p* = 0.001) (58).

In summary, various inflammatory biomarkers have been investigated, but most have been retrospective, and more evidence is needed to identify simple and cost-effective inflammatory biomarkers that can link inflammatory responses after lung cancer surgery to cancer recurrence.

## Perioperative Modulation of Inflammatory Response and Oncologic Outcome

Several perioperative factors influence the inflammatory response after surgery. Because the inflammatory status of the patient is critical for cancer recurrence, it is important for surgeons and anesthesiologists to understand these factors and apply them to perioperative management. Various perioperative management processes that modulate the inflammatory response have been studied in clinical trials, and have shown advantages in postoperative complications, but few have translated into oncological outcomes. The following section focuses on perioperative strategies that are beneficial or harmful in terms of oncological outcomes (Table 2).

**Table 2:** Perioperative factors that influence the inflammatory response after surgery and their impact on oncological outcomes.

Factor	Mechanisms	Theoretical effect	Oncological outcomes (references)
VATS, RATS vs. open thoracotomy	– Damaged tissue activate inflammatory response	−	Higher OS and DFS (63, 64) Similar OS and DFS (65–68)
TIVA vs. inhalant	– Propofol inhibits the production of inflammatory cytokines and does not suppress NK cell activity – Inhalants trigger up-regulation of hypoxic inducible factors and induce immunosuppression	−	Similar OS and DFS (69)
Dexmedetomidine	– No effect on neutrophil function – Reduces the inflammatory response of macrophages	−	Lower OS (70)
Opioids	– Reduce NK cell and macrophage activity – Decrease neutrophil function and interact with pro-inflammatory cytokines	+	Lower OS and DFS (71–73) Similar OS and DFS (74)
NSAIDs	– Decrease the amount of prostaglandin E2	−	Higher OS and DFS (75) Similar OS and DFS (76)
Regional anesthesia	– Reduces stress response *via* pain control and sympathetic block – Direct anti-inflammatory effect of local anesthetic agents	−	Epidural: Similar OS and DFS (77–79) Paravertebral: Higher OS (79)
Transfusion	– Transfusion related immunomodulation	+	Lower OS and DFS (80–83)

*VATS, video-assisted thoracoscopic surgery; TIVA, total intravenous anesthesia; NK, natural killer; NSAIDs, non-steroidal anti-inflammatory drugs.*

*“+”, pro-tumor; “−”, anti-tumor.*

### Extent of Tissue Injury

The extent of tissue injury is an important factor in the inflammatory response after surgery. Tissue and organ damage that occurs during surgical manipulation results in the release of inflammatory mediators (44). For example, macrophages in the damaged skin release chemokines such as keratinocyte chemoattractant and macrophage inflammatory protein-2, causing neutrophil infiltration. Adipocytes can secrete TNF-α, suggesting that adipose tissue damage can lead to the release of inflammatory mediators.

Clinical trials have shown that minimally invasive video-assisted thoracoscopic surgery (VATS) attenuates the inflammatory response and maintains immune cell function compared to open thoracotomy. In studies that compared VATS and thoracotomy in patients undergoing lobectomy, lower serum IL-6 and CRP levels were reported in patients who underwent VATS (84–86). A study that evaluated IL-6 levels in the pleural fluid following lobectomy also demonstrated that the increase in pleural IL-6 levels 3 h after surgery was significantly lower after VATS compared to open lobectomy (87). Similar results were reported by a study of bronchoalveolar lavage (BAL). In a study that evaluated cytokines from BAL in the contralateral lung, IL-6, IL-8 and IL-10 levels were lower in VATS patients (88). It has also been reported that VATS results in a lesser decrease in circulating T and NK cells, lesser suppression of lymphocyte oxidation, and decreased phagocyte reaction oxygen species (ROS) generation compared to open thoracotomy (85, 89–91).

VATS shows either superior or non-inferior oncological outcomes compared to conventional open thoracotomy. A retrospective study of stage IA NSCLC patients who underwent lobectomy demonstrated that VATS was associated with increased 5-year OS compared to open thoracotomy (100% vs. 87%, *p* = 0.01) and DFS (100% vs. 86%, *p* = 0.03) (63). In tumors greater than 5 cm, VATS was associated with greater OS and DFS (*p* = 0.056 and 0.031, respectively) compared to open thoracotomy (64), and in clinical N2 lung cancer, VATS showed similar 5-year OS (50.5% vs. 48.4%, *p* = 0.127) and DFS (60.5% vs. 44.6%, *p* = 0.069) (65). A study of oncological outcomes in patients who underwent surgical resection for early-stage lung cancer found no differences in 5-year OS (71.6% vs. 65.9%, *p* = 0.36) and DFS (75.2% vs. 69.2%, *p* = 0.55) between VATS and open lobectomy (66). In two National Cancer Database analyses, VATS was not found to be inferior to the open approach in terms of 5-year OS in stage I (66.3% vs. 65.8%, *p* = 0.92) and II (49.0% vs. 51.2%, *p* value not provided) NSCLC patients (67, 68). Some controversy exists because the restricted instrument handling in VATS can make complete oncologic resection difficult, despite the benefits of VATS, such as less tissue injury. Previous studies have mainly focused on comparing different surgical methods without considering the impact of perioperative management. Therefore, single-center studies with relatively consistent perioperative management might show superior oncological outcomes, while multi-center studies with various perioperative management might show noninferior oncological outcomes.

### Perioperative Medicine

#### Anesthesia Techniques

##### Anesthetic Agents

Inhaled anesthetics may promote growth of residual cancer cells by two mechanisms: upregulation of hypoxia-inducible factor (HIF) and immunosuppression. Inhaled provide organ protection in different models of organ damage, particularly in ischemia-reperfusion injury. Their protective properties are due to HIF-1α upregulation, which has been linked to more aggressive cancer phenotypes and poorer clinical prognosis (92). It has been proposed that the cytoprotective properties of HIFs in organs may also provide protection to residual cancer cells. In an *in vitro* study, isoflurane upregulated HIF-1α and HIF-2α levels and intensified VEGF expression (93). Immunosuppressive properties of inhaled anesthetics have been demonstrated in *in vitro* studies, where isoflurane and sevoflurane inhibited T lymphocytes (94), and induced apoptosis of T and B lymphocytes (95). In a study of the human NSCLC cell-line, sevoflurane suppressed NK cell cytotoxicity and increased immunosurveillance mediators (96).

Propofol, the most commonly used intravenous anesthetic agent, has anti-inflammatory effects, which may protect against perioperative immunosuppression. In lipopolysaccharide (LPS)-activated macrophages, exposure to a therapeutic concentration of propofol significantly reduces the levels of LPS-enhanced IL-1β, IL-6 and TNF-α proteins (97). Propofol can reduce inflammatory responses in LPS-induced alveolar epithelial type-III cell injury through downregulation of CD-14 and TLR-14 expressions (98, 99). In a mouse model with d-galactosamine/LPS induced acute liver injury, propofol inhibited the production of inflammatory cytokines and oxidative stress-related factors. In a study of the effects of anesthetics on NK cell activity and metastasis in a breast cancer mouse model, propofol was reported as the only agent that did not suppress NK cell activity or increase metastasis (100). Other intravenous agents such as thiopental, etomidate and ketamine, also have anti-inflammatory properties (101), and thiopental and ketamine were associated with suppressed NK cell activity (100).

Comparison of anesthetic agents have focused on the two most commonly used anesthetic agents; inhalants and propofol. Inhalants seem to attenuate the local pulmonary inflammatory response more than propofol, while propofol provides greater protection against the systemic inflammatory response. A meta-analysis of 8 studies involving 488 patients undergoing lung resection with one-lung ventilation (OLV), found no significant differences in the concentrations of systemic IL-6, IL-10 and TNF-α between sevoflurane and propofol (102). However, in the same meta-analysis, IL-6 levels in the BAL fluid in both the dependent and independent lung were decreased with sevoflurane compared to propofol (102). A prospective study reported decreased number of T lymphocytes and NK cells after surgery in NSCLC patients, but the decrease was lesser in patients who received combined sevoflurane-epidural anesthesia compared to those who received total intravenous anesthesia (103). Whether these effects on cellular immunity were due to differences between sevoflurane and propofol, or due to epidural anesthesia is unknown. Despite the different effects on cellular immunity, the 3-year DFS was similar between the two groups. Similarly, another prospective study demonstrated that postoperative serum VEGF and TGF-β levels were significantly lower in patients who received propofol and paravertebral blocks than those who received sevoflurane (104). Although inhalants and propofol seem to affect the inflammatory and immune responses differently, this has not translated into oncological outcomes. In a retrospective study of NSCLC patients who underwent curative resections, there were no significant differences in the hazard ratios for recurrence (HR: 1.310, 95% CI, 0.841, 2.041; *p* = 0.233) and death (HR: 0.902, 95% CI, 0.643, 1.265; *p* = 0.551) between sevoflurane and propofol (69).

##### Local Anesthetic Agents and Regional Anesthesia

Local anesthetic agents have beneficial effects on the inflammatory response and cancer recurrence by attenuation of the stress response and immunosuppression while reducing perioperative doses of opioids and other anesthetic agents (44). Local anesthetic agents are capable of inhibiting adhesion, chemotaxis, phagocytosis, and the production of superoxide anion and hydrogen peroxide by neutrophils and macrophages (105). Studies have shown that lidocaine can enhance NK cell activity (106). An *in vitro* study that used clinically relevant concentrations of lidocaine and ropivacaine demonstrated that local anesthetic agents inhibit TNF-α-induced invasion of lung adenocarcinoma by blocking the activation of Akt and focal adhesion kinase (107). Another *in vitro* study that used lidocaine, ropivacaine, and chloroprocaine demonstrated that amide-linked local anesthetic agents inhibited TNF-α-induced Src-activation and ICAM-1 phosphorylation, which are important in the migration of lung adenocarcinoma cells (108). In a study of pigs undergoing lung resection surgery, continuous intravenous lidocaine infusion resulted in decreased TNF-α levels in BAL, plasma, and lung samples (109). In a study of NSCLC patients undergoing VATS, patients who received intravenous lidocaine had lower serum IL-17 and cortisol compared to patients without lidocaine administration (110). Although intravenous administration of local anesthetic agents has been shown to attenuate the inflammatory response, this effect has not translated into oncological outcomes. However, the effects of local anesthetic agents through regional anesthesia have been extensively studied.

Regional anesthesia with thoracic epidural, thoracic paravertebral, intercostal nerve, or fascial plane blocks is considered an essential component of pain management in lung cancer surgery (111). It can reduce the inflammatory response to surgery and thus, cancer recurrence through several mechanisms. Regional anesthesia attenuates the stress response to surgery *via* pain control or sympathetic blocks, reduces the need for anesthetic agents, and exhibits direct effects by absorption of local anesthetic agents, as described above (112, 113). In a prospective study, patients who underwent lung cancer surgery with thoracic epidural anesthesia had significantly lower IL-6 levels in serum and lung epithelial lining fluid (114). Another prospective study of patients undergoing radical resections for lung cancer demonstrated that T lymphocytes levels were better preserved in patients who received intravenous anesthesia with epidural anesthesia compared to those who received only intravenous anesthesia (115). However, these effects did not correlate with oncological outcomes. A prospective study in patients who underwent VATS lung cancer resection demonstrated that epidural anesthesia-analgesia did not improve OS (HR: 1.12, 95% CI, 0.64, 1.96; *p* = 0.697) and DFS (HR: 0.90, 95% CI, 0.60, 1.35; *p* = 0.608) compared to general anesthesia (77). A retrospective study that compared epidural and intravenous analgesia in stage I NSCLC patients undergoing lung resections found no statistically significant differences in five-year OS (HR: 0.91, 95% CI, 0.58, 1.41; *p* = 0.663) and DFS (HR: 1.11, 95% CI, 0.12, 10.11; *p* = 0.925) (78). Another retrospective study that compared epidural, paravertebral, and intravenous patient-controlled analgesia (PCA) in patients undergoing open thoracotomy for curative resections of primary lung cancer demonstrated that pain-control methods were not related to cancer recurrence, but that paravertebral PCA may have a beneficial effect on OS (HR against epidural: 0.58, 95% CI, 0.39, 0.87; HR against PCA: 0.60, 95% CI, 0.45, 0.79; *p* = 0.002) (79).

##### Non-Intubated Thoracic Anesthesia

Mechanical ventilation is commonly used with general anesthesia in many surgeries, and OLV is used in almost all lung cancer surgeries to facilitate surgical exposure. However, mechanical ventilation and OLV are associated with a profound systemic and local inflammatory response in both ventilated and collapsed lungs (116).

Non-intubated (NI) thoracic anesthesia is a novel technique where acute lung injury and accompanying inflammatory response can be attenuated by avoiding of general anesthesia and mechanical ventilation. Conventional thoracic anesthesia is performed under general anesthesia with mechanical ventilation and OLV, whereas NI thoracic anesthesia is performed under sedation and regional anesthesia with spontaneous ventilation (117). NIVATS is a safe and feasible technique for lung cancer resection surgery (118–120). In a study of patients who underwent VATS, NIVATS resulted in an attenuated stress response compared to conventional intubated VATS (121). A study that compared NIVATS with epidural anesthesia demonstrated that NIVATS resulted in a lesser decrease in postoperative serum NK cells and total lymphocyte count compared to general anesthesia with OLV (122). In a study of patients undergoing VATS metastasectomy, NIVATS was associated with a lesser reduction of serum NK cells at 7 days after the procedure, and lesser spillage of IL-6 at 1, 7, and 14 days compared to intubated VATS (123). In a study of stage I NSCLC patients undergoing surgical resections, postoperative serum IL-6, TNF-α, and IL-6/IL-10 ratio were significantly lower in NIVATS patients compared to intubated patients (117). Although NI thoracic anesthesia has been shown to attenuate the inflammatory response, there have been no clinical studies to investigate the oncological outcomes in lung cancer surgery.

#### Opioids

Opioids are generally considered as immunosuppressive and reduce NK cell cytotoxicity and macrophage and neutrophil phagocytosis (124). They also decrease the neutrophil production of ROS, impair neutrophil chemotaxis, and decrease cytokine production (125). Interestingly, opioids can interact with inflammatory cytokines such as IL-1, IL-4, IL-6 and TNF-α, which regulate gene expression at the mu-opioid receptor to cause immunosuppression (126). In a retrospective study of stage I–III lung adenocarcinoma patients, higher intraoperative morphine administration was associated with worse OS (HR: 1.09, 95% CI, 1.02, 1.17; *p* = 0.010), whereas ketamine was associated with improved DFS (HR: 0.44, 95% CI, 0.24, 0.80; *p* = 0.007) (71). This was also demonstrated in a retrospective study of NSCLC patients undergoing VATS lobectomy, where increased doses of opioids during the initial 96 h postoperatively was associated with a higher 5-year recurrence rate (OR: 1.003, 95% CI, 1.000, 1.006; *p* = 0.04) (72). A retrospective study of NSCLC patients undergoing surgery reported that opioids were a risk factor for OS in stage I patients (HR: 1.15, 95% CI, 1.01, 1.32; *p* = 0.036), but not for stage II (HR: 0.94, 95% CI, 0.76, 1.16, *p* = 0.586) and stage III patients (HR: 0.98, 95% CI, 0.83, 1.15, *p* = 0.862) (73). However, another retrospective study of NSCLC patients undergoing curative resection reported that the amount of opioid usage did not affect the risk for recurrence (*p* = 0.521) and death (*p* = 0.660) (74).

#### Adjuvant Agents

Dexmedetomidine is known for its anti-inflammatory properties. It has no effects on neutrophil chemotaxis, phagocytosis, or superoxide production at clinically relevant doses, and it reduces the inflammatory response of macrophages (127). It can reduce the extent of lung injury by inhibiting IL-6 and TNF-α expression in lung tissues (128). A study of lung cancer patients undergoing radical resections demonstrated that dexmedetomidine reduced the inflammatory response and oxidative stress response, evidenced by lower IL-6, IL-8, and malondialdehyde levels, and higher superoxide dismutase levels compared to controls (129). In a prospective study of patients undergoing thoracoscopic surgery, intraoperative dexmedetomidine administration reduced serum HMGB-1, monocyte chemoattractant protein 1, neutrophil elastase, and IL-6 levels compared to saline infusion (130). On the other hand, dexmedetomidine has been shown to promote tumor metastases by inducing myeloid-derived suppressor cells that have immunosuppressive and pro-angiogenic properties (131). Consequently, the anti-inflammatory properties of dexmedetomidine failed to show benefits in cancer recurrence. An animal study demonstrated that dexmedetomidine increases tumor-cell retention and growth of metastases in breast, colon, and lung cancers (132). In a study of stage I–IIIa NSCLC patients undergoing surgery, intraoperative dexmedetomidine administration had no significant impact on DFS (HR: 1.18, 95% CI, 0.91, 1.53; *p* = 0.199), but was associated with worse OS (HR: 1.28, 95% CI, 1.03, 1.59; *p* = 0.024) (70).

Nonsteroidal anti-inflammatory drugs (NSAIDs) are commonly used perioperatively for their analgesic and opioid-sparing properties (101). They have well-recognized anti-inflammatory and anti-thrombotic properties. NSAIDs inhibit COX-1 and COX-2 expression, which decreases the amount of available PGE_2_. PGE_2_ upregulates the immunosuppressive IL-10, downregulates the antiangiogenic IL-12, and has a role in tumor invasion, apoptosis resistance, and dendritic cell differentiation and migration (133, 134). COX-2 can also trigger various cellular inhabitants favoring the tumor microenvironment, such as IL-1b, TGF-β, and VEGF (134). Not only do NSAIDs inhibit COX enzymes, but their opioid-sparing and anti-thrombotic properties provide a defense against cancer recurrence (135). In a retrospective study of NSCLC patients undergoing surgery, postoperative NSAID administration was related to longer OS (HR: 0.528, 95% CI, 0.278, 0.884; *p* = 0.006) and DFS (HR: 0.557, 95% CI, 0.317, 0.841; *p* = 002) (75). Meanwhile, another retrospective study of NSCLC patients undergoing surgical resections reported that postoperative NSAID administration was not an independent predictor of OS (*p* = 0.18) and DFS (*p* = 0.66) (76).

#### Transfusion

Transfusion of blood products may result in a transient depression of the immune system referred to as transfusion-related immunomodulation (TRIM) (33). TRIM may develop due to suppression of cytotoxic cell and monocyte activity, release of immunosuppressive prostaglandins, inhibition of IL-2 production, and increase in suppressor T-cell activity. Although the leukocyte reduction technique has been used to eliminate white cells implicated in TRIM, a few remaining leukocytes may still modulate the immune response in the recipient.

The concentration of cytokines is increased in stored packed red blood cell (PRC) units (136). PRC units contain pro-inflammatory lysophosphatidylcholines (lyso-PCs), which modulate NK and T cell activity, and induce pro-inflammatory cytokine production in macrophages (137). Ecosanoids, such as prostaglandins and thromboxane, can also accumulate in PRCs (138). These mechanisms all contribute to the immunomodulatory effects of PRCs, leading to a pro-inflammatory and immunosuppressive state.

Many clinical studies have investigated the role of blood transfusions in lung cancer recurrence. In a retrospective study of stage I–III NSCLC patients who were transfused for hemoglobin levels < 8.0 g/dL within 7 days after surgical resection, patients who received transfusions were at greater risk for early recurrence (HR: 1.81, 95% CI, 1.59, 2.06; *p* < 0.001) and all-cause mortality (HR: 2.38, 95% CI, 1.97, 2.87; *p* < 0.001) (80). A meta-analysis of 23 studies with 6473 patients showed that allogeneic blood transfusions were significantly associated with earlier recurrence and worse OS in patients with surgically resected lung cancers (81). In another meta-analysis of 18 studies with 5915 patients, perioperative blood transfusion was associated with worse OS (HR: 1.42, 95% CI, 1.20, 1.69; *p* < 0.001) and DFS (HR: 1.49, 95% CI, 1.29, 1.65; *p* < 0.001) in patients with resected lung cancers (82). A retrospective study of NSCLC patients who underwent pulmonary resections demonstrated that, although a single-unit blood transfusion did not affect survival, greater units of blood transfusions were associated with significantly decreased OS (2 units HR: 1.55, 95% CI, 1.262, 1.91; *p* < 0.001; 3–7 units HR: 2.02, 95% CI, 1.61, 2.53; *p* < 0.001; and ≥8 units HR: 4.29, 95% CI, 2.91, 6.33; *p* < 0.001) and DFS (2 units HR: 1.44, 95% CI, 1.19, 1.76; *p* < 0.001; 3–7 units HR: 1.85, 95% CI, 1.49, 2.30; *p* < 0.001; and ≥8 units HR: 3.57, 95% CI, 2.45, 5.21; *p* < 0.001) in a dose-dependent manner (83).

## Conclusions

The inflammatory response during cancer resection surgery is closely linked to postoperative oncological outcomes. Many factors, such as tissue injury, perioperative medications, and perioperative management (transfusion, methods of mechanical ventilation, and so forth), modulate the inflammatory response in lung cancer surgery. However, only a few high-quality clinical trials have investigated the impact of perioperative strategies on lung cancer recurrence compared to other types of cancers. Most published studies are retrospective, or prospective but designed for outcomes other than cancer recurrence. Fortunately, high-quality randomized trials are recently starting to get published (77, 139), and are in progress; “Volatile Anaesthesia and Perioperative Outcomes Related to Cancer: The VAPOR-C Trial” (NCT04316013) is evaluating propofol versus sevoflurane in colorectal or lung cancer patients (140), “General Anesthetics in CAncer REsection Surgery (GA-CARES) Trial” (NCT03034096) is evaluating propofol versus volatile agents in various types of cancer including lung cancer, and “The Effect of Combined General/Regional Anesthesia on Cancer Recurrence in Patients Having Lung Cancer Resections” (NCT02840227) is in progress.

Although results on the inflammatory response and other postoperative outcomes seem promising, current evidence does not support a change in anesthetic practice, or the use of specific agents or techniques for the purpose of reducing the risk of cancer recurrence in lung cancer surgery. Considering the enormous impact of lung cancer in the field of medicine, understanding the mechanisms of inflammation and cancer recurrence, and influencing the perioperative factors is of paramount importance. Certain anesthetic and adjuvant agents, regional anesthesia, transfusions, and NI thoracic anesthesia appear promising. Mechanical ventilation and OLV result in profound systemic and local inflammatory response in both ventilated and collapsed lung (116). Lung-protective ventilation may be a useful strategy to mitigate acute lung injury (111). As demonstrated by a study of patients undergoing open thoracic surgery, where reduction of tidal volume during OLV reduced alveolar concentrations of TNF-α and ICAM-1 (141), lung-protective ventilation might have a role in preventing cancer recurrence after lung cancer surgery. The effect of neoadjuvant therapy on perioperative inflammation during lung cancer surgery also deserves attention. A preoperative inflammatory state is associated with OS and DFS in patients undergoing neoadjuvant chemoradiotherapy followed by surgical resection (142). Preoperative chemotherapy in lung cancer patients can exacerbate the perioperative overproduction of inflammatory cytokines (143). More recently, neoadjuvant immunotherapy, using immune checkpoint inhibitors, is gaining attention for the treatment of advanced NSCLC (144). However, there is lack of data on the effects of neoadjuvant therapy on perioperative inflammation, and the association between the perioperative inflammatory state after neoadjuvant therapy and oncological outcomes. Well-designed prospective studies are needed to determine whether these perioperative management processes could contribute to better oncological outcomes. Meanwhile, the recently published guidelines for an enhanced recovery program after thoracic surgery include components that reduce surgical stress and the resultant inflammatory response (111).
